# Differential Effects of Self‐Initiated, Externally Triggered, and Passive Movements on Action‐Outcome Processing: Insights From Sensory and Motor‐Preparatory Event Related Potentials

**DOI:** 10.1111/ejn.70236

**Published:** 2025-09-04

**Authors:** Edward Ody, Tilo Kircher, Yifei He, Benjamin Straube

**Affiliations:** ^1^ Department of Psychiatry and Psychotherapy University of Marburg Marburg Germany

**Keywords:** efference copy, ERP, forward model, readiness potential, self‐initiated movement

## Abstract

Self‐initiated voluntary actions are different from externally triggered or passive movements. However, it remains unclear how these movements affect action feedback processing and how they are prepared. Here, we focus on the sensory and motor‐preparatory event‐related potentials. Participants made active (self‐initiated, 700 ms lower limit), quick (respond to a cue as quickly as possible), and passive (finger moved by device) button presses that triggered visual stimuli. The active and quick conditions elicited lower visual N1‐P2 peak‐to‐peak amplitudes than the passive condition but did not significantly differ from each other. For prestimulus ERPs (lateralized/readiness potential; L/RP), all conditions showed a negative shift in RP, with lower amplitudes in the quick than in the active condition. There were no significant differences between active and passive. For the LRP, the active and quick conditions showed a sharp deflection shortly before the button press. The amplitude of both conditions was significantly lower than passive around 100 ms before the movement. Our results suggest that active and quick movements involve similar feedback prediction, even though they are prepared differently. They thus offer a finer‐grained specification of the efference copy mechanism.

## Graphical abstract

We investigated ERPs for active (self‐initiated), passive (finger dragged down by electromagnet), or quick (as fast as possible) movements, followed by visual stimuli. In sensory ERPs, active and quick movements showed reduced peak amplitudes relative to passive. Premovement readiness potentials (RP) differed between active and quick, suggesting differences in high‐level motor preparation. Lateralized RP appeared similar across active and quick, suggesting similar motor‐specific preparation. This suggests that while the movements involved different preparation, subsequent processing of action consequences was similar.

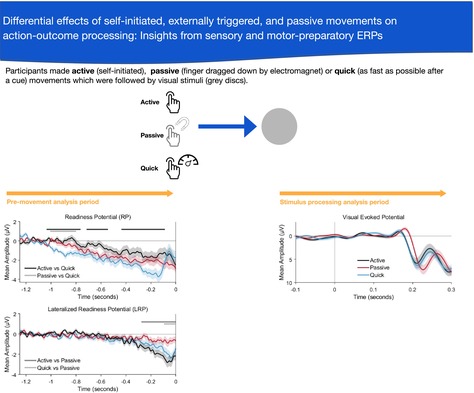

**Abbreviations**
ANOVA: analysis of variance
BF: Bayes factor
ERP: event‐related potential(s)
IQR: interquartile range
LRP: lateralized readiness potential
RP: readiness potential
TMS: transcranial magnetic stimulation
VEP: visual evoked potential

## 1 Introduction

In order to successfully interact with the environment, humans must be able to produce different types of movement, both to act on our internal intentions, goals, and desires (self‐initiated movements) and in response to events that happen around us (externally cued movements). For example, when driving, you might decide to push the accelerator to overtake another car (self‐initiated). Conversely, if another vehicle swerves into your lane without looking, you might need to hit the brake pedal to avoid a collision (externally cued). Despite their relevance to everyday life, the underlying neurobiology between these movements is not clear.

Though the output of these movement types (in this example, pushing a pedal) might appear similar from a behavioral standpoint, imaging studies suggest that they involve dissociable brain networks. Self‐initiated actions recruit greater activity in frontal midline and posterior parietal areas than externally cued actions (Jahanshahi et al. 1995; Jenkins et al. 2000). With higher temporal precision, prior EEG studies have primarily focused on movement preparation, and they suggest distinct motor preparation mechanisms for self‐initiated and externally cued movements. Preparation for self‐initiated movements begins up to several hundred milliseconds before the execution of the movement and may include preparation of the goal, trajectory, and timing of the movement (Haggard 2008). This period is accompanied by a slowly rising negative cortical potential in the scalp‐recorded EEG (called the Readiness Potential; RP) (Kornhuber and Deecke 2016), typically maximal in fronto‐central electrodes. A similar but reduced potential also precedes externally cued movements provided that the cue can be anticipated (Thickbroom et al. 1985; Jahanshahi et al. 1995).

Self‐initiated and externally cued movements may also differ in action‐outcome prediction. When preparing to move, the brain sends a copy of the motor command (efference copy; von Holst and Mittelstaedt 1950) to the relevant sensory cortices. This signal represents a prediction for the sensory outcome of the action. When the action is executed, the actual sensory feedback is compared to what was predicted, and if they match, the sensory response (neural and behavioral) to the action feedback is modulated (Martikainen et al. 2005; Baess et al. 2011; Sanmiguel et al. 2013; Straube et al. 2017; Arikan et al. 2019; Press et al. 2020; Fuehrer et al. 2022; Lubinus et al. 2022; Ody, Straube, et al. 2023; Ody et al. 2024). Previous EEG studies have typically found reduced sensory responses (in the form of lower early N1/P2 peak amplitudes) for self‐compared to externally‐generated sensations (for example, Martikainen et al. 2005; Bäß et al. 2008; Baess et al. 2011; Sanmiguel et al. 2013; Ody, Straube, et al. 2023), assumed to result from additional efference copy‐based predictive processes.

Although self‐initiated and externally cued movements may differ in terms of preparation time, intention, and efference copy‐based prediction, the question remains how they affect the processing of their sensory consequences. With few exceptions, previous studies investigating self‐ and externally‐generated sensations have not considered different types of movement. Typically, these studies had participants produce movements within an approximate time constraint (for example, once every few seconds) but did not take other aspects of the movement into account. Some studies have included involuntary movements (i.e., produced by TMS, nerve stimulation, or other devices) (for example, Timm et al. 2014; Jack et al. 2021; Ody, Straube, et al. 2023). They showed suppression of early ERP components elicited by the self‐generated stimuli compared to those triggered by the involuntary movements, suggesting that movement preparation is necessary for sensory modulation. However, does the sensory processing of an action's outcome differ as a function of the movement type (e.g., self‐initiated vs. externally cued)? It is likely that for both movements, the differential levels of motor preparation and intention may directly impact sensory processing. For example, at the behavioral level, Juravle and Spence (2012) showed that perception of vibrotactile feedback differs between self‐initiated and externally cued movements. Alternatively, as both movements are nevertheless voluntary and accompanied by motor preparation activity (efference copy), they might induce comparable predictive mechanisms that lead to similar processing of their sensory outcome. Gaining insight into this remaining issue will provide us with a finer‐grained understanding of the efference copy mechanism.

Therefore, this study aimed to investigate the neural correlates of self‐initiated and externally cued movements, and more importantly, the behavioral consequence and neural underpinning for processing their elicited sensory feedback. What exactly defines a self‐initiated movement and the distinction between self‐initiated and externally triggered movements is a major point of discussion in the literature (Pfister et al. 2012; Haggard 2019). In this experiment, we made an analogous distinction to Obhi et al. (2009) and Pfister et al. (2012) by considering self‐generated actions to be those not made in response to an external cue and externally triggered actions to be those made immediately in response to an external imperative stimulus. Self‐initiated (henceforth active) movements were self‐paced (except for a 700 ms lower limit so that participants would not press the button immediately in response to the cue; see Section 2.2 for details). Externally cued movements (henceforth quick) were made as quickly as possible in response to an auditory cue. We also included involuntary (henceforth passive) movements, which were produced using a device that dragged the participant's finger down. The button presses were followed by a visual stimulus. We investigated how these different movements affected the processing of their sensory consequences and how premovement activity differed between them. As participants had the most temporal control over the active movements, we expected that there would be greater movement preparation and therefore more negative RP amplitudes associated with them. Likewise, we expected that the passive condition would have the lowest RP amplitude. Following the same logic, we hypothesized that the active condition would have the lowest early sensory ERP (N1 and P2) amplitudes (after subtracting motor‐related activity recorded in motor‐only control conditions), followed by the quick and then the passive condition.

When a unimanual movement is made, there is strongly lateralized activity, typically shortly before the movement is executed. This lateralization can be measured by subtracting the ipsilateral from the contralateral activity, forming a component known as lateralized readiness potential (LRP) (Smulders and Miller 2012). LRP was generally not investigated in prior studies on externally cued movements. However, there is evidence that it is reduced before involuntary (passive) movements (Ody, Kircher, et al. 2023; Ody et al. 2024). As the LRP is related to motor‐specific preparation that occurs shortly before a movement, we expected that it would be present before both active and quick movements and absent before the passive ones. However, due to the lack of previous literature, we did not hypothesize whether or not the active and quick conditions would differ.

## 2 Method

### 2.1 Participants

Twenty‐four participants took part in the study, of which 16 were female. Ages ranged between 18 and 34 (M = 24.96, SD = 4.53). Participants were recruited through a university mailing list and received a €20 inconvenience allowance for taking part. By self‐report, all were right‐handed, had normal or corrected‐to‐normal hearing and vision, no history of drug or alcohol abuse, no history of serious brain injury, and no first‐degree relatives with Schizophrenia. Participants were naïve to the purpose of the experiment. The study protocol was approved by the local ethics committee in accordance with the Declaration of Helsinki (except for preregistration) (World Medical Association 2013), and all participants provided written informed consent.

### 2.2 Stimuli and Procedure

Participants read written instructions and then completed a training exercise consisting of 10 trials from each experimental condition (active, passive, and quick). During the training, the “active” and “passive” blocks were always presented first, followed by the “quick” training block, but the order of “active” and “passive” was counterbalanced across participants.

The experiment was completed in a dimly lit room. Participants sat at a comfortable distance behind a 19″ computer monitor running at 60 Hz. The right hand was placed on a button pad with the right index finger loosely attached to the button with elastic fabric. The left hand was placed on the computer keyboard on which the response keys (“v” and “n”) were marked. Participants wore earplugs and headphones. Pink noise was played through the headphones throughout the experiment to mask mechanical sounds produced by the button, which can potentially influence results (Horváth et al. 2012; Horváth 2015). Before starting the main experiment, the volume of the noise was adjusted to a comfortable level for each participant. The volume level of the auditory tone cue was also adjusted to ensure the participant could hear and respond to it. The experiment was presented with Psychtoolbox V 3.0.12 (Brainard and Vision 1997; Pelli 1997) running on GNU Octave V 4.0.0 (Eaton et al. 2025) in Linux.

Participants completed three types of trials. All trial types had an auditory cue to signal that the button could be pressed. In active trials, a 700 ms lower limit was imposed on the button presses. This was done to elicit a well‐prepared self‐initiated movement that was not merely a response to the cue. Previous studies have used similar methods (Rohde and Ernst 2012; van Kemenade et al. 2016, 2017). If the button was pressed before 700 ms, the message “Too quick. Try again” was displayed, and the trial was repeated. In the passive trials, the tone signalled that the button would be dragged down by the button box. The duration between the tone and button press was recorded in each trial in the active condition, and these values were shuffled and used in the passive condition. To avoid extended waiting periods in the passive condition, button presses that took longer than 2.5 s were given a value of 2.5 s. Latencies exceeding 2.5 s were removed from all further analyses. In the quick trials, participants were told to press the button as quickly as possible after hearing the cue. The mean (standard deviation) reaction times in the three conditions were 1128 ms (310 ms) for active, 1129 ms (331 ms) for passive, and 243 ms (216 ms) for quick. The median (interquartile range) reaction times in the three conditions were 1062 ms (375 ms) for active, 1050 ms (368 ms) for passive, and 166 ms (162 ms) for quick.

**FIGURE 1:**
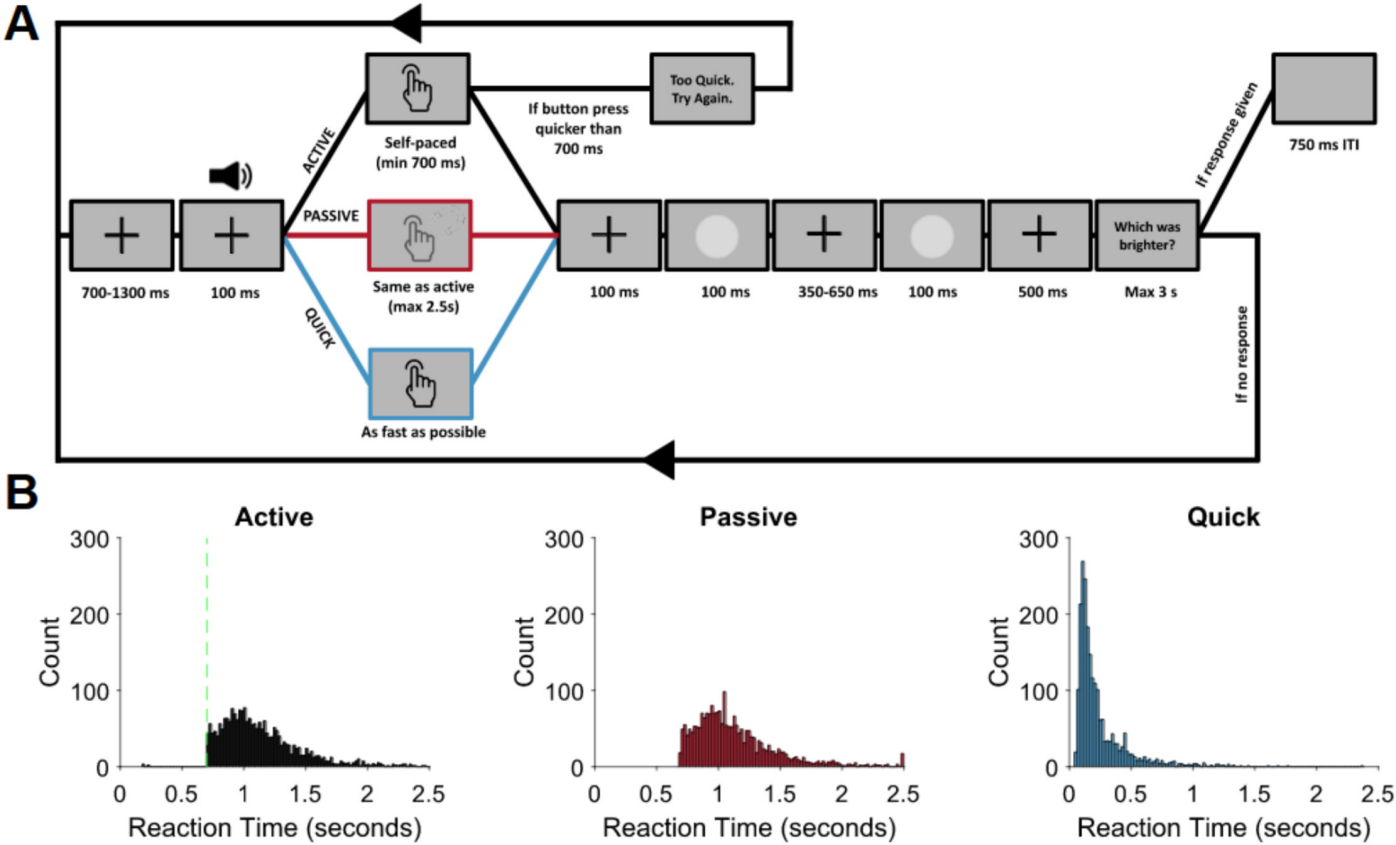
(A) Schematic illustration of the task. (B) Reaction times for the button presses measured as the time between the audio cue offset and the button press, for all experimental trials collapsed across all participants. The green marker in active shows the 700 ms lower limit on button presses in this condition. The reaction times do not include responses above 2.5 s or below 50 ms (quick condition), as these were removed before analysis (see Section 2.2).

All trials began with a fixation cross. The auditory cue (1000 Hz tone) was presented after a variable delay (700–1300 ms in steps of 100 ms) for 100 ms. After the button press was made, there was a 100 ms interval followed by a visual stimulus for 100 ms. Due to a technical error, stimuli in the passive condition were delivered one frame (approximately 16 ms) later compared to the active and quick conditions. This delay was included to improve temporal prediction of the stimulus. Active and passive movements differ in how well the onset of action effects can be predicted. Active movements have temporal control, meaning that the action effect onset is more predictable than passive movements. Adding a delay helps to reduce this difference, as both button presses can act as a cue that signals the temporal onset of the action effect. We have used the same button‐stimulus delay in other studies (Ody, Kircher, et al. 2023; Ody, Straube, et al. 2023; Ody et al. 2024). Next came a variable interstimulus interval (350–650 ms in steps of 50 ms), followed by a second, identical stimulus. Another interval of 500 ms was presented and finally the question “Which was brighter?” Participants were not informed that the stimuli had the same brightness level. The behavioral task was based on the design of Reznik et al. (2015), who implemented a similar design where participants had to judge the subjective intensity of two identical tones. Participants used the “v” key to indicate that they thought the first stimulus was brighter and the “n” key for the second stimulus. If no response was made after 3 s, the trial was repeated. Making a response triggered a 750‐ms intertrial interval. A schematic of the task is shown in Figure 1.

Trials were presented in mini‐blocks of 25 experimental trials and three catch trials. Regular visual stimuli were presented with a fixed luminance of 84.55 cd/m^2^. In the catch trials, the second stimulus had a lower luminance of 28.45 cd/m^2^. The purpose of these was to ensure participants were paying attention. Catch trials were inserted into the trial sequence at random, and they were not included in behavioral and EEG data analyses. Mini‐blocks were presented in the order of active, passive, and quick. This sequence was repeated four times, meaning that each condition had 100 experimental trials.

After the experimental blocks, participants completed a control task (henceforth motor‐only). Following standard contingent paradigm procedure (Horváth 2015), this task was designed to record EEG activity associated with the movements in isolation (without any contingent stimulus). The activity recorded in the motor‐only conditions was subtracted from the experimental conditions in the sensory ERP analysis to isolate signals related to sensory processing (see Section 2.5). Motor‐only trials had the same fixation cross, tone cue, and button press as experimental trials. However, after the button press, no stimulus was presented. Instead, the fixation cross remained on screen for a variable interval (1650–1950 ms, in steps of 50 ms). The duration was chosen to be approximately the same length as an experimental trial. After this interval had passed, the cross disappeared for 750 ms. Motor‐only trials were presented in the order active, passive, and quick. This sequence was repeated twice, and each mini‐block contained 28 trials, meaning participants completed 56 trials of each type in total.

### 2.3 EEG Data Acquisition

EEG was continuously recorded at a sampling rate of 500 Hz from 32 active Ag/AgCl electrodes (Fp1/2, F7/8, F3/4, Fz, FT9/10, FC5/6, FC1/2, T7/8, C3/4, Cz, TP9/10, CP5/6, CP1/2, P7/8, P3/4, Pz, O1/2, and Oz). The EEG was referenced online to the electrode location FCz, and the ground electrode was placed on the forehead. Impedances were kept at 25 kΩ or below. The signal was amplified by a BrainVision amplifier and recorded with BrainVision Recorder (Brain Products GmbH, Gilching Germany). Electrodes were mounted in an elastic cap (actiCAP, Brain Products GmbH, Gilching, Germany) according to the international 10–20 system.

### 2.4 EEG Preprocessing

Preprocessing was completed using the EEGLAB toolbox (Delorme and Makeig 2004) in MATLAB (R2020a Mathworks, Sherborn, Massachusetts). EEG data were downsampled to 250 Hz and re‐referenced to the average of electrodes TP9 and TP10. Line noise was removed using the Zapline Plus function (Klug and Kloosterman 2022). A high‐pass filter was applied at 1 Hz, and the data were subjected to an extended infomax ICA. Components were classified using ICLabel (Pion‐Tonachini et al. 2019), and any that were identified as muscle, eye, or channel noise with greater than 79% estimated accuracy were removed. The ICA results were then applied to the unfiltered data. Finally, the data were bandpass filtered between 0.01 and 100 Hz. Further analyses were completed with the Fieldtrip toolbox (Oostenveld et al. 2011) and custom routines in MATLAB. Trials with button press reaction times lower than 50 ms (quick condition) were removed, as such short reactions could indicate that the button was already pressed down before the audio cue was heard. Button presses longer than 2.5 s were excluded from all subsequent analyses, as this was the cutoff for the passive condition (see Section 2.2). The remaining continuous EEG then underwent different segmentation procedures for evaluating both the sensory ERPs (N1/P2) and the motor preparatory slow waves (RP and LRP).

### 2.5 Sensory ERPs (N1/P2)

EEG was segmented into epochs from 0.5 s before to 1 s after the button press. Electrodes, which had a range greater than 300 μV in more than 50% of trials (per condition), were removed and interpolated using the Fieldtrip function ft_channelrepair with spline interpolation. A single participant had 12 electrodes that met these criteria and was therefore removed from all subsequent analyses. Trials were then submitted to an additional artifact rejection routine. Those with a range greater than 250 μV and/or with large variance (greater than 10 standard deviations above or below the mean z‐transformed data) in any channel were removed. These steps were implemented with the ft_artifact_zvalue and ft_artifact_threshold fieldtrip functions. At the end of this process, one participant had conditions containing fewer than 25% of total trials and was therefore removed from all subsequent analyses.

A bandpass filter was applied between 0.5 and 20 Hz. The data were baseline‐corrected to the 100 ms period after the button press (100 ms before the first stimulus). The mean activity per participant, channel, and time point was calculated for each condition. The activity in the motor‐only conditions was then subtracted from the equivalent experimental conditions, as in other similar experiments (for example, Bäß et al. 2008; Whitford et al. 2011; Mifsud et al. 2018; Csifcsák et al. 2019).

An average of electrodes Oz, O1, and O2 was used for analysis. For statistical analysis, the N1 and P2 peaks were extracted. The peaks of these components were visually determined, and then the minimum (N1) or maximum (P2) value within a suitable time window (140–200 ms after button press for N1, and 200–240 ms after button press for P2) was identified separately for each condition. A 16‐ms time window was defined around this center point, and a mean amplitude was calculated for each participant in this range. N1‐P2 peak‐to‐peak amplitude was also tested. This was calculated by subtracting the mean P2 peak amplitudes from the mean N1 peak amplitudes. The peak‐to‐peak amplitude provides a better signal‐to‐noise ratio and has been measured in previous studies (for example, Timm et al. 2014).

### 2.6 RP and LRP

For the comparison of the RP/LRP, continuous EEG was segmented from 1.3 s before to 0.5 s after the button press and baseline‐corrected between 1.25 and 1 s before the button press. Channel and trial rejection procedures were implemented in the same way as the sensory ERPs. Unlike the sensory ERPs, the premovement data were bandpass filtered between 0.01 and 40 Hz. RP was taken as an average across electrodes Cz, C3, and C4, while LRP was derived by subtracting activity at electrode C3 from activity at electrode C4.

To provide a broader view of cortical dynamics, signals from additional electrode sites, including prefrontal (Fz, F3, and F4) and parietal (Pz, P3, and P4) regions, are presented in the Supporting Information. These are included for completeness but are not interpreted here, as the present study focuses on motor‐related preparatory activity.

### 2.7 Intensity Judgment Task

To assess performance in the behavioral task, we compared how often participants responded that the first or second stimulus was brighter, expressed as a percentage of total responses. Since the luminance of the stimulus of interest was held constant throughout the experiment, we would expect a guess to lead to an even number of responses for “first brighter” and “second brighter,” while a systematic bias towards one or the other should represent a change in perception. As in the EEG preprocessing, responses greater than 2.5 s or under 50 ms (quick trials only) were excluded. The percentage of “2nd stimulus brighter” responses was calculated for each participant and each condition.

### 2.8 Correlations

Several previous studies have found significant correlations between measures of premovement activity and sensory ERPs in contingent paradigm experiments (e.g., Ford et al. 2007, 2014). In particular, Ford et al. (2014) found that LRP amplitude in the 100‐ms interval before the button press was correlated with N1 suppression related to a tone triggered by the button press. In our experiment, LRPs had significantly greater amplitudes in the active and quick conditions than the passive condition approximately 100 ms before the button press. In the sensory ERPs, the opposite pattern was found for P2 and N1‐P2 peak‐to‐peak, with larger amplitudes in the passive condition than in the active and quick conditions. It is possible that motor preparation in this period is related to preparing for the upcoming action feedback. Therefore, we conducted a post hoc but theory‐driven correlation between LRP and P2 amplitude. We performed Pearson correlations between the mean LRP amplitude in the time window identified by the permutation test and the mean P2 peak amplitude. In a previous experiment (Ody, Straube, et al. 2023), we showed that P2 suppression was correlated with behavioral suppression measured in an intensity judgment task. Therefore, we also tested this correlation in the current data set. Due to the difference in designs, with three conditions in this experiment instead of two, we tested correlations between the mean P2 peak amplitudes and the percentage of “2nd brighter” responses in the behavioral task. We performed one correlation per condition (active, passive, and quick) and one across all conditions. The correlations were Bonferroni‐corrected for multiple comparisons, which resulted in an alpha level of *p* < 0.013 to be a significant result.

### 2.9 Details of Statistical Analysis

Permutation statistics were implemented using the permutest function (Gerber 2023) in MATLAB. Other statistical analyses were implemented using JASP (Love et al. 2019). For the Bayesian statistics, we followed the recommendations given by Keysers et al. (2020). Accordingly, Bayesian ANOVAs were conducted with default priors, and effects are reported as the Bayes factor for the inclusion of a particular effect (BFincl), calculated as the ratio between the likelihood of the data given the model with vs. the next simpler model without that effect. For post hoc pairwise comparisons, we conducted two‐tailed Bayesian *t*‐tests and report Bayes factor BF10 that represents *p* (data|H+:factor1 ≠ factor2)/*p* (data|H0:factor1 = factor2). The magnitude of Bayes factors was interpreted according to Andraszewicz et al. (2015) (i.e., anecdotal, moderate, strong, very strong, or extreme, evidence for the alternative/null hypothesis).

For sensory ERPs, mean peak amplitudes were compared across movements (active, passive, and quick) using repeated measures ANOVA and repeated measures Bayesian ANOVA. Bonferroni‐corrected post hoc *t*‐tests were performed when the ANOVA was significant.

For premovement ERPs, following earlier findings suggesting the ramping pattern of the RP (Ody, Kircher, et al. 2023), we did not average the RP/LRP within predefined time windows. Rather, we compared the conditions using cluster‐based permutation tests. Each time point was tested with a two‐tailed dependent samples *t*‐test using a significance threshold of *p* < 0.05. Contiguous time points exceeding this threshold were grouped into clusters, and the sum of the *t*‐values was used as the test statistic for the permutation test. This process was repeated 10,000 times (10,000 permutations) with shuffled condition labels to determine a distribution of the probability of observing a cluster (or clusters) with that test statistic value. Clusters within the highest or lowest 2.5th percentile were considered significant. This process was repeated for all combinations of the three conditions (active vs. passive, quick vs. passive, and active vs. quick).

For the intensity judgment task, the percentage of “2nd stimulus brighter” responses was entered into a one‐way repeated measures ANOVA and repeated measures Bayesian ANOVA with the factors of movement type (active, passive, and quick).

## 3 Results

### 3.1 ERP (N1/P2)

**FIGURE 2:**
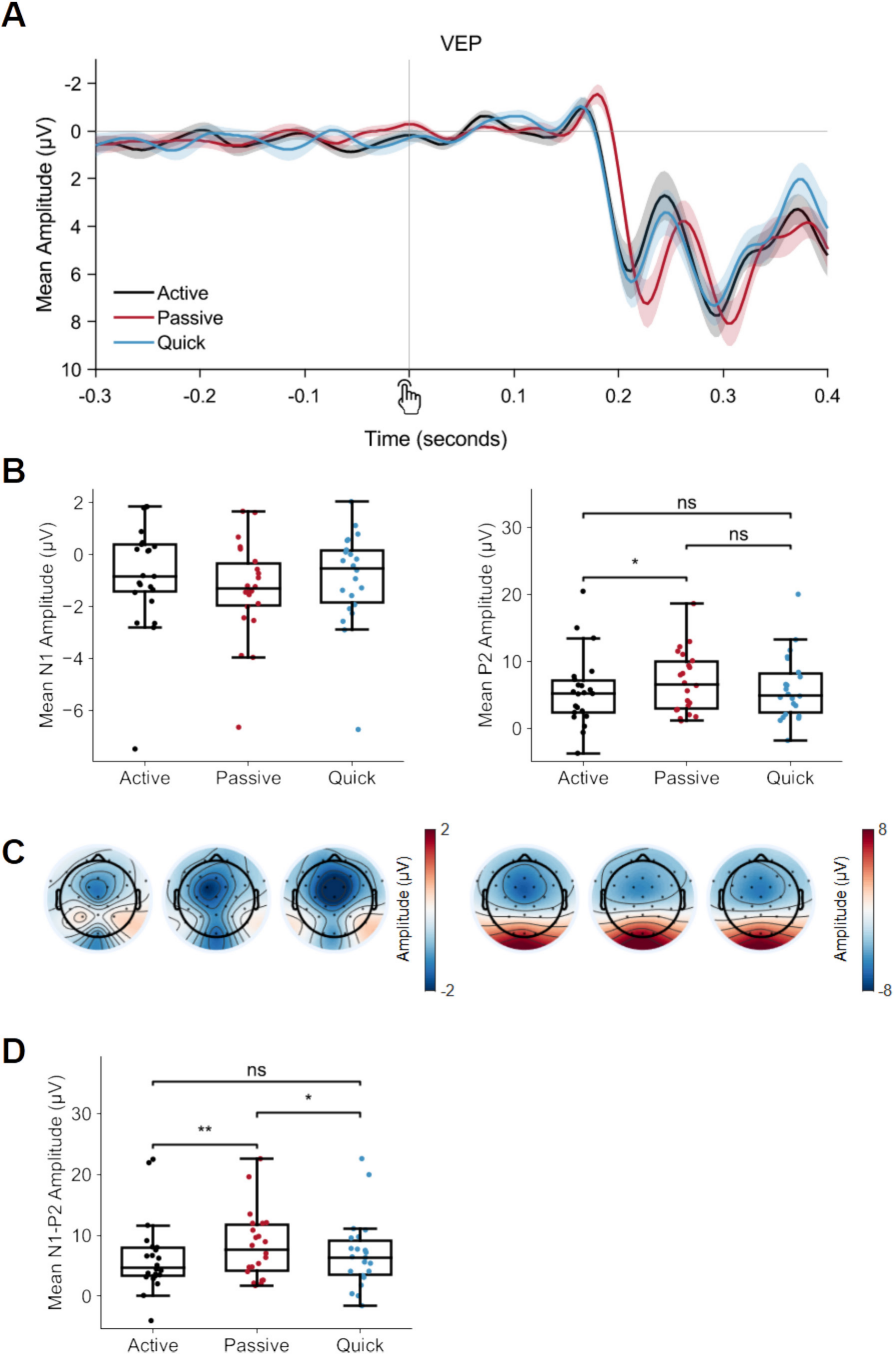
Sensory ERP results. (A) ERP waveforms for the average of electrodes Oz, O1, and O2, time‐locked to the button press with the baseline window in the 100‐ms period after the button press (−100 to 0 ms before the stimulus). Motor‐related activity recorded in the motor‐only control conditions was subtracted from the experimental conditions to derive these sensory ERPs. Shaded areas around the waveforms show the standard error. Note that the passive condition is later as stimuli in this condition were presented one frame (approximately 16 ms) later than the active and quick conditions. (B) Boxplots showing the distribution of N1 and P2 peak amplitudes. (C) Scalp topography maps averaged over the N1/P2 time windows used for analysis. (D) Boxplots showing the distribution of N1‐P2 peak‐to‐peak amplitude. *p < 0.05, **p < 0.01, ***p < 0.001.

N1/P2 results are reported in Figure 2. The selected N1 time windows (relative to the button press) were 160–176 ms for active, 172–188 ms for passive, and 156–172 ms for quick. For P2, they were 204–220 ms for active, 220–236 ms for passive, and 200–224 ms for quick. For the N1 amplitudes, the effect of movement, *F*(2, 42) = 1.07, *p* = 0.351, η_p_^2^ = 0.05, BF_incl_ = 0.28, was not significant, with moderate evidence in favor of the null hypothesis. For the P2, there was a significant effect of movement, *F*(2, 42) = 4.27, *p* = 0.021, η_p_^2^ = 0.17, BF_incl_ = 2.53, with anecdotal evidence in favor of the alternative hypothesis. Bonferroni‐corrected post hoc tests revealed a significant difference between active and passive, *t* = 2.88, *p* = 0.019, BF_10_ = 3.35 (with moderate evidence in favor of the alternative hypothesis). The difference between quick and passive was not significant, *t* = 1.88, *p* = 0.200, BF_10_ = 1.05, although there was anecdotal evidence in favor of the alternative hypothesis. The difference between active and quick was not significant, *t* = −0.99, *p* = 0.981, BF_10_ = 0.38, with the Bayes factor suggesting anecdotal evidence in favor of the null hypothesis. In other words, the mean amplitude in the passive condition (6.97 μV) was significantly higher than the active condition (5.61 μV) and marginally higher than the quick (6.08 μV) condition, but the active and quick conditions did not significantly differ from one another. There was also a significant effect of movement in the N1‐P2 peak‐to‐peak amplitudes, *F*(2, 42) = 6.96, *p* = 0.002, η_p_^2^ = 0.25, BF_incl_ = 14.79, with strong evidence in favor of the alternative hypothesis. Post hoc tests showed a moderate significant difference between active and passive, *t* = −3.64, *p* = 0.002, BF_10_ = 9.84, a moderate significant difference between quick and passive, *t* = 2.54, *p* = 0.045, BF_10_ = 4.56, but no significant difference between active and quick, *t* = −1.1, *p* = 0.833, BF_10_ = 0.41, with anecdotal evidence in favor of the null hypothesis. The mean amplitudes were 8.38 μV for passive, 5.96 μV for active, and 6.98 μV for quick.

### 3.2 RP and LRP

**FIGURE 3:**
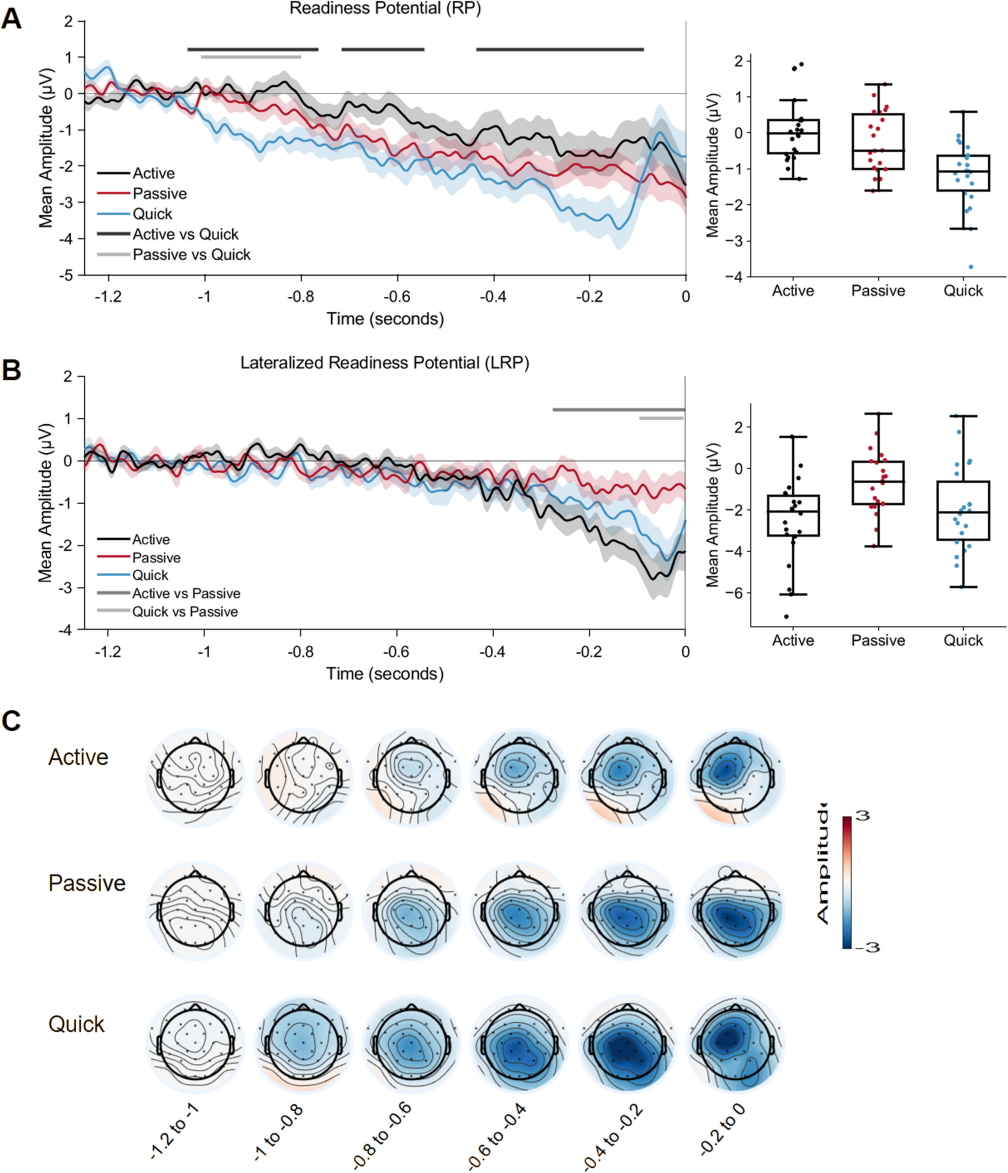
Premovement ERP results. (A, left) Readiness potential waveforms, averaged across Cz, C3, and C4 and time‐locked to button press. Baseline period 1–1.25 s before button press. (A, right) Boxplots showing the distribution of amplitudes in the early RP, where active and passive significantly differed from quick (approximately 0.8–1 s before button press). (B, left) Lateralized readiness potential as an average of C3‐C4. Baseline period 1–1.25 s before button press. (B, right) Boxplots showing the distribution of amplitudes in the early RP, where active and passive significantly differed from quick (approximately 100 ms before button press). (C) Scalp topography maps for the time series.

RP and LRP results are presented in Figure 3. For the RP, there were three clusters showing a significant difference between active and quick, with lower amplitudes in the quick condition. There was also a significant cluster showing a significant difference between passive and quick around 800–1000 ms before the button press. For the LRP, there was a significant cluster for the comparison between active and passive starting approximately 200 ms before the movement. For the comparison between quick and passive, there was a significant cluster approximately 100 ms before the movement.

### 3.3 Intensity Judgment Task

The effect of movement type on the percentage of “2nd stimulus brighter” responses was not significant, *F*(2, 42) = 0.55, *p* = 0.582, η_p_^2^ = 0.025, BF_10_ = 0.19, with moderate evidence in favor of the null hypothesis. A figure showing the behavioral results is presented in Figure S1.

### 3.4 Correlation

There were no significant correlations. Detailed statistics are presented in Table S1.

## 4 Discussion

This study investigated electrophysiological correlates of different types of movement and their sensory consequences. Participants were given a cue and then had to either wait for at least 700 ms before making a button press (active), wait for a device to move their finger (passive), or respond as quickly as possible (quick). Our results suggest that the efference copy mechanism might work similarly for both self‐initiated and externally cued movements, despite the fact that movement initiation, intention to move, task demand, and preparation time differ between conditions. This is indicated by a similar pattern of feedback processing (N1‐P2 suppression) and LRPs. However, premovement RPs did differ between the active and quick conditions, suggesting that participants need to prepare differently to achieve comparable perceptual processing for the feedback of the two movement conditions.

In the sensory‐related ERPs, N1‐P2 peak‐to‐peak amplitudes were lower in the active and quick conditions than in the passive condition. The active and quick conditions did not show significant differences. The visual ERPs, elicited by the button press‐triggered stimuli, revealed lower N1‐P2 peak‐to‐peak amplitudes for the active and quick conditions than for the passive condition (Bäß et al. 2008; Knolle et al. 2013; Sanmiguel et al. 2013; Timm et al. 2013; Oestreich et al. 2015; Elijah et al. 2016). In the visual domain, the direction of the effect has been mixed, although the majority of studies show a modulation effect of self‐initiation (Schafer and Marcus 1973; Gentsch and Schütz‐Bosbach 2011; Hughes and Waszak 2011; Mifsud et al. 2018; Csifcsák et al. 2019; Ody, Straube et al., 2023). In a previous study (Ody, Straube, et al. 2023), we showed that stimuli triggered by active button presses elicited smaller N1 and P2 peak amplitudes than stimuli triggered by passive movements, supporting the notion of sensory suppression of visual action consequences. However, of note, in this previous study, we did not control the timing between the cue and the button presses for the passive condition as we did in the current study. Here, we observed again a voluntary (active/quick) vs. involuntary (passive) modulation, at least for the N1‐P2 amplitudes. This data pattern thus corroborates the idea that efference copy‐based predictions, as induced by voluntary actions, lead to reduced processing of visual action outcomes. Notably, we did not observe an N1 difference between the active and the passive conditions. This might be related to the controlled preparation timing between both conditions, which was not implemented in the earlier study. However, it is worth considering that the quick condition, which had quicker response times, also did not show an N1 difference when compared to the passive condition. Nevertheless, further within‐subject studies are necessary to investigate whether the timing of the active movements has an impact on early sensory components such as the N1 and the P2.

Most importantly, there was evidence that sensory processing did not differ between the active and quick conditions, in spite of the differences in RP (see below). This finding might indicate that both types of movement are accompanied by comparable efference copy‐based predictions, even though they are prepared differently (see, for example, Jahanshahi et al. 1995; Obhi and Haggard 2004). This result stands in contrast to Juravle and Spence (2012), where a differential behavioral consequence was observed for both movements. It should be noted, however, that the two designs used very different movements and tested different sensory modalities. Furthermore, as this is a null result, it should be interpreted with some caution. Future studies should consider incorporating different movement types and sensory modalities to gain a finer‐grained understanding of premovement prediction mechanisms.

The RPs showed a slow negative drift in all three conditions, suggesting that all the movements were anticipated. Contrary to our expectations, the permutation test showed lower amplitudes in the quick condition than in the active condition across almost the entire analysis window. This result contrasts with Jahanshahi et al. (1995) who found lower amplitudes for self‐initiated button presses compared to externally cued ones. These differences are likely due to differences in the design of the two studies. In the present experiment, participants saw a fixation cross at the start of the trial, followed by an audio cue after a mean interval of 1 s. The quick condition, where participants were asked to respond as quickly as possible to an audio cue, is similar to Contingent Negative Variation (CNV) experiments (Walter et al. 1964; Brunia et al. 2012). These experiments have a warning stimulus, which signals an imperative stimulus after some time duration. During this period, participants are preparing to move (like a runner waiting for the starting pistol at the beginning of a race). We conducted an additional analysis time‐locked to the onset of the fixation cross (see Supporting Information). This analysis confirmed that there was a more negative amplitude for the quick condition than for the active and passive conditions preceding the cue, suggesting that this preparation indeed took place. In Jahanshahi et al. (1995), the cues were yoked to the rate of the self‐initiated movements, potentially making them more unpredictable and therefore more difficult to prepare for several hundred milliseconds in advance.

In motor‐based paradigms, the RP and CNV are difficult to dissociate, as they both precede movements and share similar scalp distribution (Brunia et al. 2012). CNV amplitude is shown to be greater for fast compared to slow movements (e.g., Rohrbaugh et al. 1976). Trovò et al. (2021) have also shown that RP amplitude scales with time pressure (i.e., imperative to move), with larger amplitudes for shorter time limits. Our results are consistent with this, as the quick condition had the shortest time limit (i.e., as fast as possible), meaning the strongest imperative to move.

It is unexpected that there were no significant differences between RP amplitudes for active and passive. This result stands in contrast to a previous result (Ody, Kircher, et al. 2023), where we found differences between active and passive in the RP. Two potentially important differences between the two studies were the timing of the button presses and the 700 ms lower limit. Passive button presses were temporally matched to the active condition in the current study but had fixed values in the previous study. This, combined with the 700 ms lower time limit, could serve to make the passive button presses more predictable. Participants were not told precisely what the 700 ms lower limit for button presses was, but it is possible that they gained a representation through trial and error in the active condition. If this is the case, it would mean that participants were able to anticipate the passive button presses, with the probability of a press occurring at any one moment increasing throughout this period and being very certain once the entire period has elapsed (the median for passive button presses was 1050 ms, only 350 ms after the 700 ms lower limit). Button press times were also clustered closely around the median (IQR = 368 ms), meaning that the press was likely to occur in the period shortly after the 700 ms limit had elapsed.

In summary, the present pattern of results showed the quick condition standing apart from the active and passive conditions. This could be due to task differences in preparation (stronger imperative to move, more attention required, etc.), irrespective of the voluntariness of the movement.

For the LRP, there was a sharp negative deflection in the active and quick conditions around 200 ms before the button press that was not present in the passive condition. LRP is related to the motor‐specific preparation that occurs shortly before a movement, originating in the primary motor cortex (M1), due to its lateralization over motor electrodes (Brunia et al. 2012; Smulders and Miller 2012). It follows logically that LRP should be reduced before involuntary movements, because there is no motor‐specific preparation for such movements, even if there is higher‐level anticipation of the movement. In a previous study (Ody, Kircher, et al. 2023), we tested this assumption by comparing self‐paced movements with ones made by the same passive movement device as we used here, and we observed reduced LRP (i.e., less negative) for passive movements. This result is replicated in the present experiment, where only the passive condition showed reduced LRP. In spite of the differences in RP, the active and quick conditions seemed to involve similar motor‐specific preparation, even though the time limits between both movements differ. This is also consistent with Trovò et al. (2021), who observed no sensitivity of the LRP in response to their time‐limit manipulation. Together with the earlier finding, our data corroborate the motor‐specific functional interpretation of the LRP. Across both experiments, regardless of time limits, LRP is only sensitive to the voluntariness of the movement.

Behaviorally, while previous similar studies have shown suppression (Reznik et al. 2015; Lubinus et al. 2022) or enhancement (Reznik et al. 2015) in intensity judgment tasks, we observed suppression for all conditions, but no significant differences between the conditions and therefore no evidence in support of suppression or enhancement related to self‐initiation. Furthermore, unlike our previous experiment (Ody, Straube, et al. 2023), we did not find evidence that P2 amplitude was correlated with behavioral responses. However, there were differences in the design of both studies, such as the duration of the stimulus presentation, the luminance of the stimulus, the variability of stimulus luminance, and the average duration of the interstimulus interval. The LRP pattern was consistent with the sensory ERP pattern, with the two conditions showing greater motor‐specific preparatory activity (active and quick) also being associated with lower sensory responses. However, unlike Ford et al. (2014), we did not find evidence of a correlation between these two measures.

As previously stated, the active movements in this study were defined as movements that are not made in response to an external cue (Obhi et al. 2009; Pfister et al. 2012). There has been extensive discussion regarding what constitutes a truly volitional movement (Haggard 2008, 2019). Haggard (2008) considers only movements that are entirely stimulus‐independent to be volitional. They should depend on an internal decision about whether to act, which action to perform, and when to do so without any external influence. In the laboratory context, many studies paradoxically ask participants to “act freely,” for example, when they have the urge to move (Jahanshahi et al. 1995; Cunnington et al. 2002; Wiese et al. 2004). However, such movements could not be considered volitional under this strict definition (Khalighinejad et al. 2018). Previous studies have also operationalized self‐initiated and externally cued movements differently. For example, Juravle and Spence (2012) had participants throw or catch a ball. In Khalighinejad et al. (2018), participants could choose between waiting for a signal (potentially for a long interval) and receiving a larger reward or skipping the waiting period and receiving a smaller reward. Other studies have compared self‐paced movements to movements synced to a periodic external signal (Toyomura et al. 2012; e.g., Taniwaki et al. 2013). Of these studies, only Juravle and Spence (2012) measured sensory responses. In the present study, control of the timing of the movements was a major difference between the active and quick movements. Future studies could investigate the effect of other movement manipulations on the action's outcome, for example, perceptual decision‐making (as implemented by Khalighinejad et al. 2018).

### 4.1 Limitations

A potential limitation of the study design is that the audio cue could have influenced the RP results. The “quick” button presses were naturally performed much closer to the auditory cue than the active and passive conditions, meaning that auditory activity was present in the waveforms at different latencies during the premovement time period. The response times for the quick condition also had a lower dispersion than the active and passive conditions, meaning that the auditory activity was less likely to be cancelled out by averaging out noise. In the “quick” RP waveform (Figure 2), an ERP peak is visible around 200 ms before the button press, which likely reflects auditory activity related to the cue. Active and passive button press response times had higher dispersion; therefore, this activity was likely cancelled out. No peak is easily identifiable in the grand average waves in Figure 2. It is unlikely that this drastically influenced the results, as the CNV (time‐locked to the audio cue; Figure S2) shows a similar pattern emerging even before the onset of the cue. Nevertheless, there will be some differences between the conditions inherent to the design, and we therefore advise caution in interpreting the results. As the cue was presented binaurally, the subtraction procedure used to derive the LRP should cancel out any auditory activity; therefore, these results are not affected.

Another limitation of the current study is that the stimuli in the passive condition had a slightly longer delay (approximately 16 ms) than in the active and quick conditions. Two previous studies have shown that suppression of self‐generated tones is affected by delay, with longer delays eliciting lower levels of suppression (Oestreich et al. 2016; Pinheiro et al. 2019). Though the additional passive stimulus delay was short, it is possible that the differences in amplitude between the active and quick conditions and the passive condition would have been larger without it.

## 5 Conclusion

For the first time, this study compared the EEG correlates of motor preparation and processing of the action's sensory outcome between three movement types, which differ in their degree of voluntariness and the imperative to move. For sensory perception, both the active condition (where participants were free to choose when to move, except for a 700 ms lower limit) and quick condition (where participants were asked to respond as quickly as possible) showed reduced amplitudes relative to the passive condition (where the participant's finger was moved by a device). This result aligns with a vast body of previous studies showing suppression of self‐generated action consequences and is consistent with the idea of an efference copy‐based forward model mechanism that predicts self‐generated action feedback. However, we did not find evidence that the active and quick movements differed in their feedback processing. This suggests that the efference copy mechanism might work similarly for both self‐initiated and externally cued movements, despite the fact that movement initiation, intention to move, task demand, and preparation time differ between conditions. Premovement RPs did differ between the active and quick conditions, suggesting that participants prepared differently for the two movement types. However, motor‐specific preparation as shown by the LRP appeared similar across both movement types. Our results thus provide novel empirical evidence for a better understanding of the functional interpretation of the premotor EEG potentials and offer a finer‐grained specification of the efference copy mechanism.

## Author Contributions

**Edward Ody:** conceptualization, data curation, formal analysis, investigation, methodology, software, validation, visualization, writing – original draft. **Tilo Kircher:** conceptualization, funding acquisition, project administration, resources, supervision, writing – review and editing. **Yifei He:** conceptualization, formal analysis, methodology, supervision, writing – review and editing. **Benjamin Straube:** conceptualization, funding acquisition, methodology, project administration, resources, supervision, writing – review and editing.

## Conflicts of Interest

The authors declare no conflicts of interest.

## Peer Review

The peer review history for this article is available at https://www.webofscience.com/api/gateway/wos/peer‐review/10.1111/ejn.70236.

## Supporting information


**Figure S1:** Behavioral results showing the percentage of responses where each participant said the 2nd stimulus in the trial was brighter for each condition.
**Figure S2:** ERPs preceding the audio cue. (A, left) Average of electrodes Cz, C3, and C4, time‐locked to the audio cue. Baseline period 800–1000 ms before the audio cue. (A, right) Boxplots showing the distribution of amplitudes in the time window where active and passive both significantly differed from quick (light gray bar in time series). (B) Scalp topography maps for the time series.
**Figure S3:** Premovement waveforms, averaged across Fz, F3, and F4 and time‐locked to button press. Baseline period 1–1.25 s before button press.
**Figure S4:** Premovement waveforms, averaged across Pz, P3, and P4 and time‐locked to button press. Baseline period 1–1.25 s before button press.
**Table S1:** Correlation analyses results.

## Data Availability

The data that support the findings of this study are available from the corresponding author upon reasonable request.
