# Supplementary material for: Differential Effects of Self‐Initiated, Externally Triggered, and Passive Movements on Action‐Outcome Processing: Insights From Sensory and Motor‐Preparatory Event Related Potentials
Source: Eur J Neurosci. 2025 Sep 4;62(5):e70236. doi: 10.1111/ejn.70236 (PMC12411811; doi:10.1111/ejn.70236)
Supplement: Supplementary file 1 — Figure S1: Behavioral results showing the percentage of responses where each participant said the 2nd stimulus in the trial was brighter for each condition. Figure S2: ERPs preceding the audio cue. (A, left) Average of electrodes Cz, C3, and C4, time‐locked to the audio cue. Baseline period 800–1000 ms before the audio cue. (A, right) Boxplots showing the distribution of amplitudes in the time window where active and passive both significantly differed from quick (light gray bar in time series). (B) Scalp topography maps for the time series. Figure S3: Premovement waveforms, averaged across Fz, F3, and F4 and time‐locked to button press. Baseline period 1–1.25 s before button press. Figure S4: Premovement waveforms, averaged across Pz, P3, and P4 and time‐locked to button press. Baseline period 1–1.25 s before button press. Table S1: Correlation analyses results. [file EJN-62-0-s001.pdf]

Supplementary Materials

1 Behavioural Results

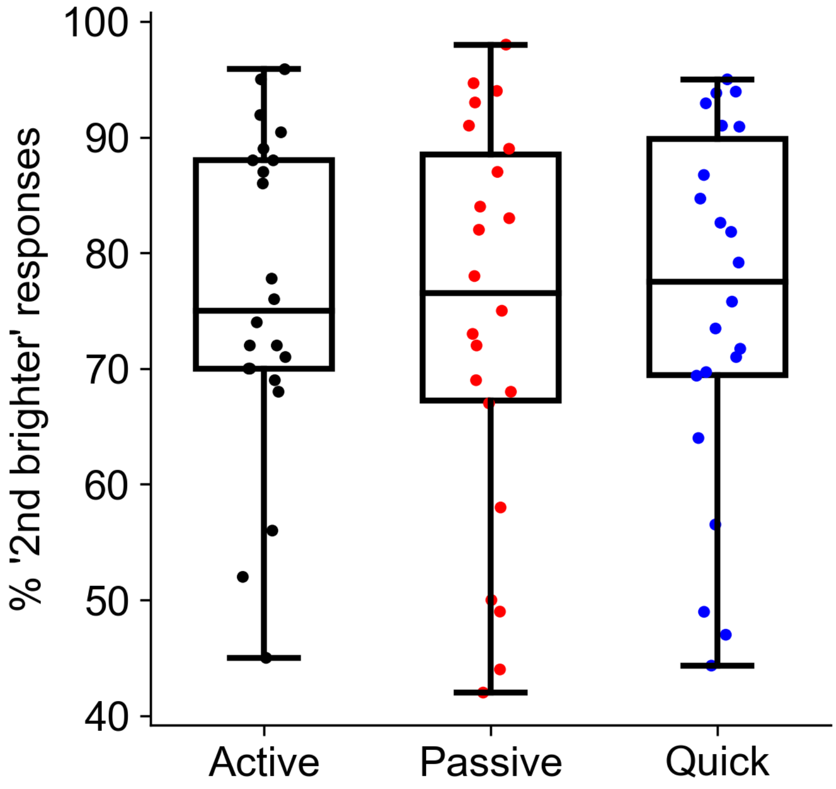

Figure S1. Behavioural results showing the percentage of responses where each participant said the 2nd stimulus in the trial was brighter for each condition.

2 Contingent Negative Variation

Contrary to our hypothesis, the quick condition had more negative RP amplitude than the active condition. This could be due to the design we used, where the onset of the fixation cross at the start of the trial is followed by an audio cue after an interval of approximately 1 second. For the quick condition, we asked participants to respond to this cue as quickly as possible, meaning that this design is similar to those used to elicit contingent negative variation (CNV) (Brunia *et al.*, 2012). During this period, participants would therefore have been preparing to make the button press. However, in the active condition, participants needed to wait to make a response after hearing the cue (and perhaps even suppress an immediate response to the cue), meaning that preparing to respond during the fixation cross-cue interval was not as necessary.

We tested whether this factor could have affected the readiness potential results by examining ERPs in the period leading up to the cue. Data were preprocessed as described in the EEG preprocessing section. They were segmented between 1 second before and 0.5 seconds after the cue and baseline corrected to the period between 1 second and 800 ms before the cue. We then took an average of electrodes Cz, C3 and C4. The waveforms were

tested using the same cluster-based permutation test procedure described in the Readiness Potential section of the main text.

The permutation tests showed that the quick condition had a robustly lower amplitude than the active and passive conditions starting around 300 ms before the cue. This suggests that participants were preparing to make the quick responses during this period. These results are shown in Figure S2.

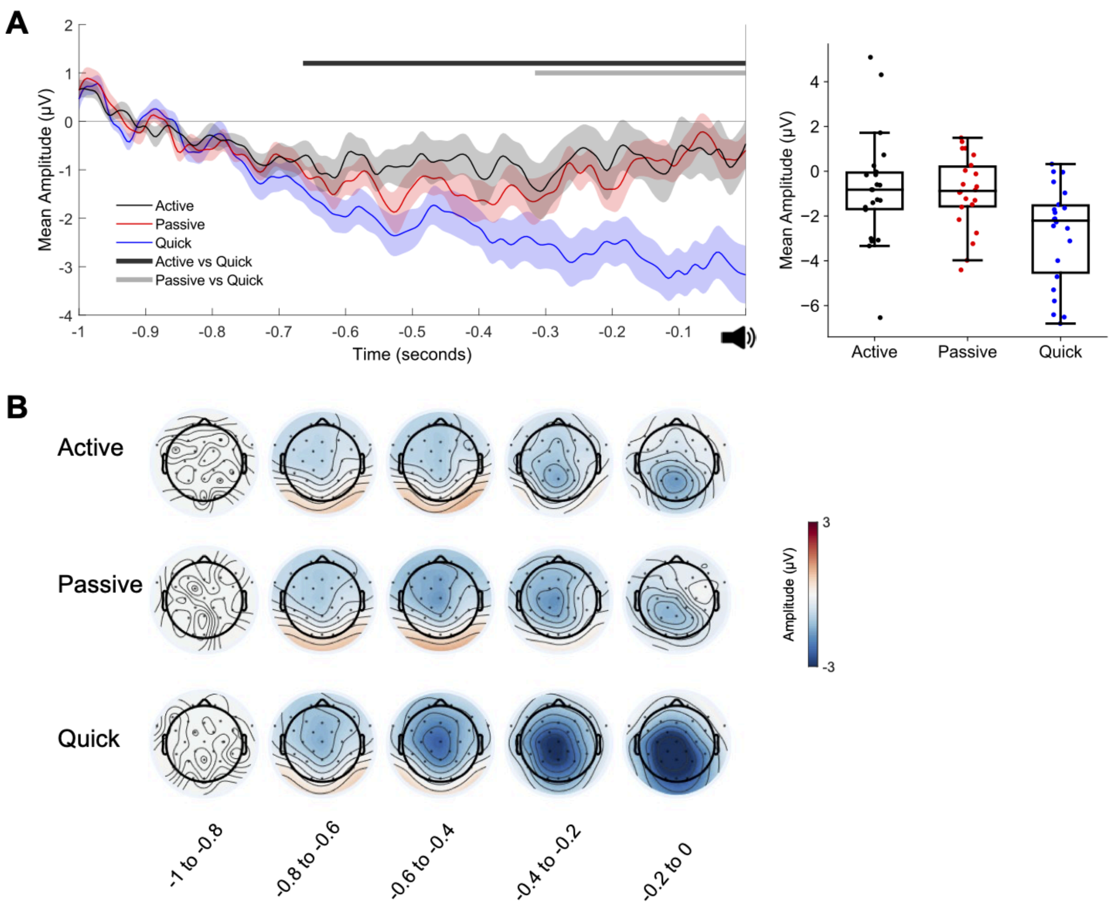

Figure S2. ERPs preceding the audio cue. (A, left) Average of electrodes Cz, C3 and C4, time-locked to the audio cue. Baseline period 800-1000 ms before the audio cue. (A, right) Box plots showing the distribution of amplitudes in the time window where active and passive both significantly differed from quick (light grey bar in time series). (B) Scalp topography maps for the time series.

3 Additional Pre-Movement Analysis

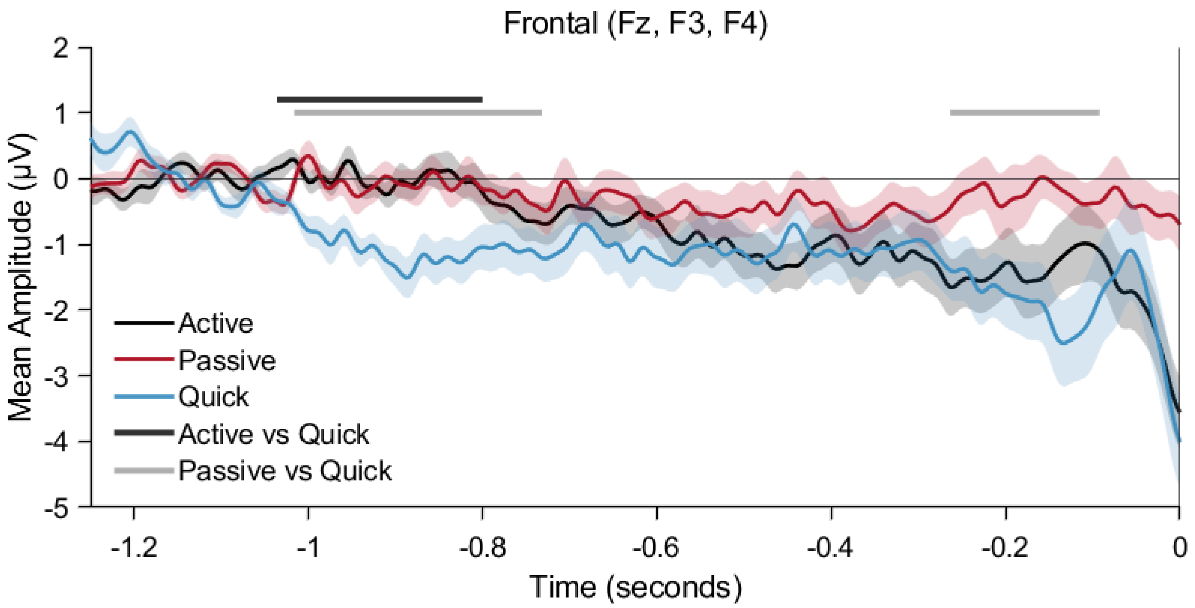

Figure S3. Pre-movement waveforms, averaged across Fz, F3 and F4 and time-locked to button press. Baseline period 1-1.25 seconds before button press.

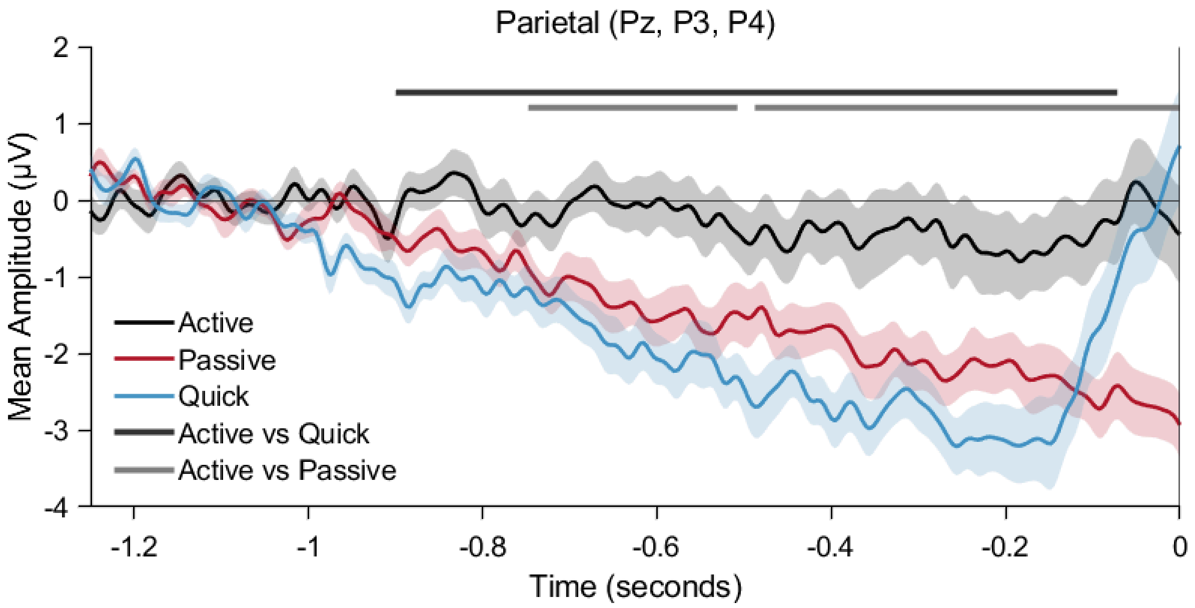

Figure S4. Pre-movement waveforms, averaged across Pz, P3 and P4 and time-locked to button press. Baseline period 1-1.25 seconds before button press.

4 Correlations

Table 1 shows the correlation results. Note that the p values displayed are uncorrected.

Table 1. Correlation analyses results.

Condition 1	Condition 2	*r*	*p*
Active P2	Active LRP	-0.19	.400

Amplitude	Amplitude		
Passive P2 Amplitude	Passive LRP Amplitude	-0.27	.228
Quick P2 Amplitude	Quick LRP Amplitude	-0.22	.336
P2 Amplitude (All Conditions)	LRP Amplitude (All Conditions)	-0.15	.213
Active P2 Amplitude	Active % ‘2nd Brighter’	0.25	.258
Passive P2 Amplitude	Passive % ‘2nd Brighter’	0.15	.506
Quick P2 Amplitude	Quick % ‘2nd Brighter’	0.26	.234
P2 Amplitude (All Conditions)	% ‘2nd Brighter’ (All Conditions)	0.22	.072

References

Brunia, C.H.M., van Boxtel, G.J.M., & Böcker, K.B.E. (2012) Negative Slow Waves as Indices of Anticipation: The Bereitschaftspotential, the Contingent Negative Variation, and the Stimulus-Preceding Negativity. *The Oxford Handbook of Event-Related Potential Components*, 1–22.
